# The course of facial corticobulbar tract fibers in the dorsolateral medulla oblongata

**DOI:** 10.1186/s12883-021-02247-z

**Published:** 2021-05-31

**Authors:** Takamichi Kanbayashi, Masahiro Sonoo

**Affiliations:** Department of Neurology, Teikyo University School of Medicine, Kaga 2-11-1, Itabashi-ku, Tokyo, 1738605 Japan

**Keywords:** Corticobulbar tract, Facial paresis, Lateral medullary infarction, Medulla oblongata

## Abstract

**Background:**

The course of the corticobulbar tract (CBT) to the facial nucleus has been investigated by some previous studies. However, there are some unclear points of the course of the CBT to the facial nucleus. This study aimed to elucidate the detailed course of the CBT to the facial nucleus through the analysis of lateral medullary infarction (LMI) cases.

**Methods:**

The neurological characteristics and magnetic resonance imaging findings of 33 consecutive patients with LMI were evaluated. The location of the lesions was classified rostro-caudally (upper, middle, or lower) and horizontally. Further, we compared the neurological characteristics between the groups with and without central facial paresis (FP).

**Results:**

Eight (24%) patients with central FP ipsilateral to the lesion were identified. Dysphagia and hiccups were more frequently observed in the group with central FP than in the group without central FP. In patients with central FP, middle medullary lesions and those including the ventral part of the dorsolateral medulla were more frequently observed. Contrastingly, patients with lesions restricted to the lateral and dorsal regions of the dorsolateral medulla did not present with central FP.

**Conclusion:**

The results of this study indicate that the CBT to the facial nucleus descends with the corticospinal tract at least to the middle portion of the medulla, and then ascends to the facial nucleus through the medial and ventral areas of the dorsolateral medulla after decussation.

## Background

The functional anatomy of the facial motor pathway is believed to be well known, but actually there remain some unclear points regarding the course of the corticobulbar tract (CBT) to the facial nucleus. Previous studies have shown that at least some CBT fibers to the facial nucleus descend to the medulla, then cross to the contralateral side, and finally ascend towards the facial nucleus located in the caudal pons [1, 2]. Some cases of lateral medullary infarction (LMI), i.e., Wallenberg syndrome, are actually accompanied by central facial paresis (FP) [3–5]. However, this neural pathway is rarely described in neuroanatomical textbooks [6–8], and the precise localization of the CBT to the facial nucleus within the medulla oblongata remains unclear. Although previous researchers have reported that the CBT to the facial nucleus mainly reaches the upper medulla [1, 2], the level to which this neural pathway descends has not been fully elucidated.

In this study, we aimed to clarify the course of the CBT to the facial nucleus within the medulla oblongata by analyzing the symptoms and detailed lesion location of LMI.

## Methods

We extracted the records of patients diagnosed with LMI from the inpatient database of Teikyo University from January 2010 to March 2020. Board-certified neurologists evaluated the neurological findings of these patients. The presence or absence of FP and whether FP was central or peripheral were investigated from the medical records. Patients with a previous history of FP and those with other intracranial lesions that might cause FP were excluded. All patients underwent brain magnetic resonance imaging (MRI), and the detailed location of the lesion was confirmed on diffusion-weighted and fluid-attenuated inversion recovery images of MRI obtained within 2 weeks of onset.

**Fig. 1:**
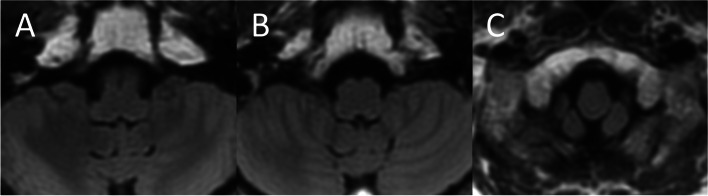
Classification of the lesion in the rostro-caudal direction on brain magnetic resonance imaging. **a** Upper medulla: the level with an inferior cerebellar peduncle bulging outward. **b** Middle medulla: the level with an outward bulging due to the inferior olivary nucleus. **c** Lower medulla: the level presenting a round shape, and without an outward bulging due to the inferior olivary nucleus. These images are the authors’ original work

**Fig. 2:**
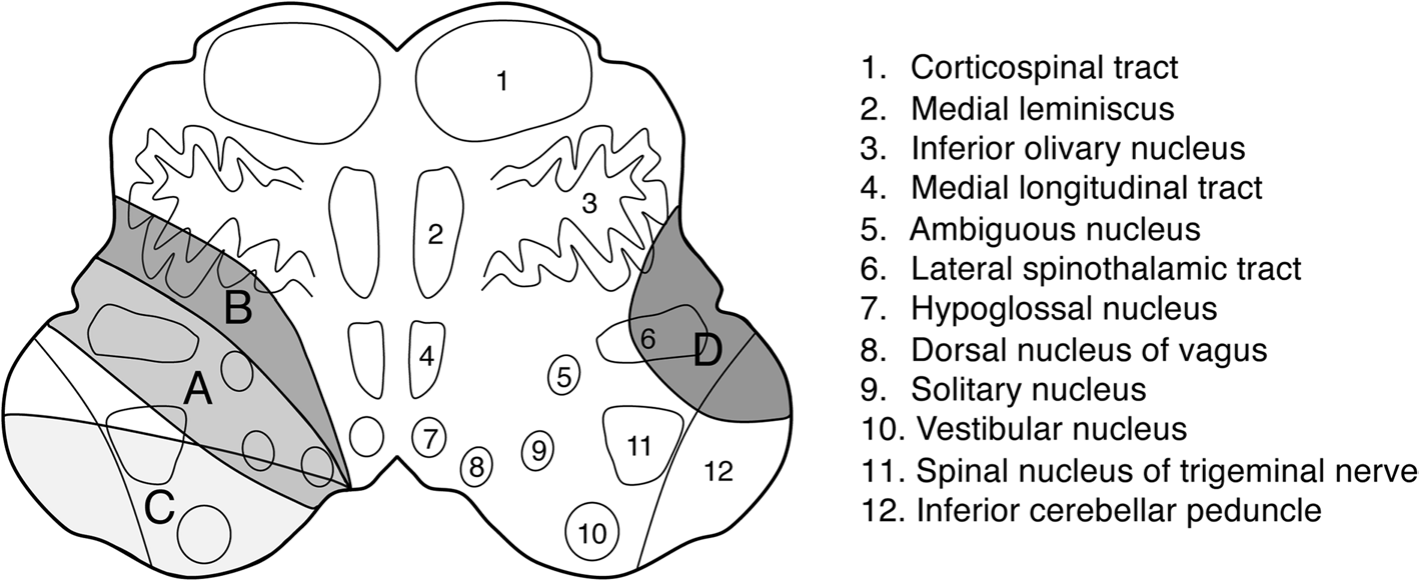
A schematic representation of the classification of the lesion in the horizontal direction. The major anatomical structures at the level of the inferior olivary nucleus and classification of the lesion are described. A, ventral type; A + B, expanded ventral type; C, dorsal type; D, lateral type; A + B + C, extensive type

Based on the previous studies, the location of the lesions was classified according to rostro-caudal and horizontal directions [9]. In the rostro-caudal direction, the lesion was categorized at three different levels as follows (Fig. 1): a) the upper medulla, characterized by massive bulging of the dorsolateral area due to the inferior cerebellar peduncle; b) the middle medulla, characterized by outward bulging due to the inferior olivary nucleus; and c) the lower medulla, characterized by a relatively round shape without outward bulging due to the inferior olivary nucleus. An extensive lesion spreading to two levels was classified as upper to middle or middle to lower. Regarding the localization in the horizontal section, the lesion was categorized into five types as follows (Fig. 2): 1) ventral type: band-shaped lesions sparing the most dorsolateral portion; 2) expanded ventral type: although similar to the ventral type, lesions expanding ventrally, and involving a part of the inferior olivary nucleus; 3) dorsal type: lesions restricted to the dorsal medulla; 4) lateral type: lesions restricted to the lateral side without involvement of the dorsal medulla; 5) extensive type: large lesions involving the dorsal medulla with area of the expanded ventral type. For each classification of lesions, the presence or absence of FP and whether FP was central or peripheral were investigated.

The other neurological symptoms and signs that may be caused by LMI, including vertigo, nystagmus, Horner’s sign, dysarthria, dysphagia, hiccups, hoarseness, impairment of pain and thermal sensations, limb ataxia, and gait ataxia were also evaluated. We statistically analyzed the differences in the percentage of lesion location and neurological findings between the groups with and without central FP.

Fisher’s exact test was used for statistical analysis. All statistical analyses were performed using JMP software version 14.1.0 (SAS Institute Inc.). A *p* value < 0.05 was considered to be statistically significant. The investigation was approved by the ethics committee of Teikyo University (Approval No. 19–220).

## Results

Thirty-eight patients with LMI were identified during the study period. One of them had a previous history of FP, and four patients had other intracranial lesions that could cause FP. Finally, 33 patients with LMI were enrolled in this study. They consisted of 25 men and 8 women, aged from 34 to 87 (mean 64.8 ± 14.9) years. The lesion was on the right side in 18 (55%) patients. Eight (24%) patients presented with central FP and two (6%) patients presented with peripheral FP. Moreover, all FP were observed on the same side as the lesion. Central FP observed in this study was mild in all cases, and there were no cases in which it remained as a sequela at the time of discharge.

The localizations of the lesions are summarized in Table 1. In the rostro-caudal classification, lesions of the middle medulla were the most frequent (52%). In the horizontal classification, the lesions of the dorsal and lateral type were the most frequent (27% for each type) and only one patient (3%) showed an expanded ventral type lesion. All patients with lesions of the lower medulla were classified as the lateral type in the horizontal classification.

**Table 1:** Lesion location of patients with lateral medullary infarction

Lesion classification	All (*n* = 33), (%)	With central FP (*n* = 8), (%)	Without central FP (*n* = 25), (%)	*p* value*
*Rostro-caudal classification*				
Upper	4 (12)	0 (0)	4 (16)	n.s.
Upper/Middle	1 (3)	0 (0)	1 (4)	n.s.
Middle	17 (52)	7 (88)	10 (40)	0.024
Middle/Lower	4 (12)	1 (13)	3 (12)	n.s.
Lower	7 (21)	0 (0)	7 (28)	n.s.
*Horizontal classification*				
Ventral	8 (24)	4 (50)	4 (16)	n.s.
Expanded ventral	1 (3)	1 (13)	0 (0)	n.s.
Dorsal	9 (27)	0 (0)	9 (36)	n.s.
Lateral	9 (27)	0 (0)	9 (36)	n.s.
Extensive	6 (19)	3 (38)	3 (12)	n.s.
Including Ventral^a^	15 (45)	8 (100)	7 (28)	< 0.001
Excluding Ventral	18 (55)	0 (0)	18 (72)	

*FP* facial paresis, *n.s.* not significant

^*^ *p* value for comparison between the groups with and without central facial paresis

^a^ The lesion including the area of ventral type, i.e., ventral, expanded ventral, extensive

Among patients with central FP, in the rostro-caudal classification, 7 (88%) and 1 (13%) patients were classified in middle and middle to lower, respectively. Similarly, in the horizontal classification, 4 (50%), 1 (13%), and 3 (38%) patients were classified in ventral, expanded ventral, and extensive type, respectively. All patients with central FP had lesions including the area of ventral type. The frequency of lesions including the area of ventral type was significantly higher in the group with central FP than in the group without central FP (*p* < 0.001).

The neurological signs and symptoms of all patients are summarized in Table 2. In all the patients, impairment of pain and thermal sensations, gait ataxia, vertigo, and Horner’s sign were common, whereas hiccups and hoarseness were relatively rare. However, in the comparison of the groups of patients with and without central FP, hiccups and dysphagia were more frequently observed in the group with central FP (*p* = 0.010 for hiccups and *p* = 0.015 for dysphagia).

**Table 2:** Neurological symptoms and signs in patients with lateral medullary infarction

Symptoms and signs	All (*n* = 33), (%)	With central FP (*n* = 8), (%)	Without central FP (*n* = 25), (%)	p value*
Vertigo	25 (76)	6 (75)	19 (76)	n.s.
Nystagmus	18 (55)	5 (63)	13 (52)	n.s.
Horner’s sign	24 (73)	8 (100)	16 (64)	n.s.
Dysarthria	9 (27)	2 (25)	7 (28)	n.s.
Dysphagia	16 (48)	7 (88)	9 (36)	0.015
Hiccups	3 (9)	3 (38)	0 (0)	0.010
Hoarseness	3 (9)	0 (0)	3 (12)	n.s.
Sensory impairment	28 (85)	8 (100)	20 (80)	n.s.
Ipsilateral face/ contralateral extremities	8 (24)	3 (38)	5 (20)	n.s.
Contralateral face/ contralateral extremities	9 (27)	3 (38)	6 (24)	n.s.
Ipsilateral face	2 (6)	0 (0)	2 (8)	n.s.
Contralateral extremities	9 (27)	2 (25)	7 (28)	n.s.
Limb ataxia	21 (64)	4 (50)	17 (68)	n.s.
Gait ataxia	27 (82)	5 (63)	22 (88)	n.s.

*FP* facial paresis, *n.s.* not significant

^*^ *p* value for comparison between the groups with and without central facial paresis

## Discussion

Facial CBT fibers mainly arise from the facial area of the motor cortex and descend together with the corticospinal tract (CST). The CBT to the facial nucleus had been considered to decussate at the level of the pons. However, some previous studies have shown that at least some fibers of the CBT to the facial nucleus that innervate the lower facial muscles descend to the level of the medulla along with the CST. These fibers then ascend to the facial nucleus through the dorsolateral medulla after decussation [1, 2, 10], and impairment of this neural pathway causes central FP in patients with LMI.

Previous studies on the neurological characteristics of LMI have reported that the frequency of incidence of FP was 21% to 56% [1, 3–5, 9, 11, 12]. The frequency of FP in the current study was 24%, which was similar to that previously reported. Thus, FP is not an uncommon neurological manifestation in patients with LMI.

In this study, FP was observed on the same side of the lesion location, regardless of whether FP was central or peripheral type, and this was consistent with the findings of previous studies [3, 5]. As a mechanism of peripheral FP in LMI, involvement of the facial nucleus caused by the extension of lesions up to the caudal pons has been postulated [13]. Indeed, in this study, the lesion of the two patients with peripheral FP involved the upper medulla.

The prognosis of central FP observed in this study was good. Kim et al., reported 12 cases with central FP in their case series of LMI, and as in the present study, FP was mild in all cases [5]. These findings may indicate that only a part of the CBT to the facial nucleus descends to the medulla.

Terao et al. reported that central FP was more commonly observed in upper than in middle or lower medullary lesions, and there was only one patient who presented with central FP by middle to lower medullary lesion in their study [1]. In this study, FP was not detected in patients with lesions confined to the lower medulla, but most patients who presented with central FP showed middle medullary lesions. Our results indicate that the CBT to the facial nucleus descends at least to the level of the middle medulla. Therefore, central FP due to middle medullary lesions may be more common than previously reported.

Dysphagia and hiccups were significantly more common in the group with central FP than in the group without central FP. Dysphagia in LMI is thought to be caused by a lesion of the ambiguous nucleus [3], which is situated in the reticular formation and about halfway between the inferior olivary and spinal nucleus of the trigeminal nerve [6]. Although the relationship between the lesion location of LMI and hiccups has not been completely elucidated, it has been reported that middle medial medullary lesions were highly associated with intractable hiccups [14]. Park et al. reported that dorsolateral lesions of the middle medulla frequently induce hiccups [15]. However, the lesion sites in all the cases with hiccups that they reported included the area of ventral type lesions, when they were substituted in our classification.

**Fig. 3:**
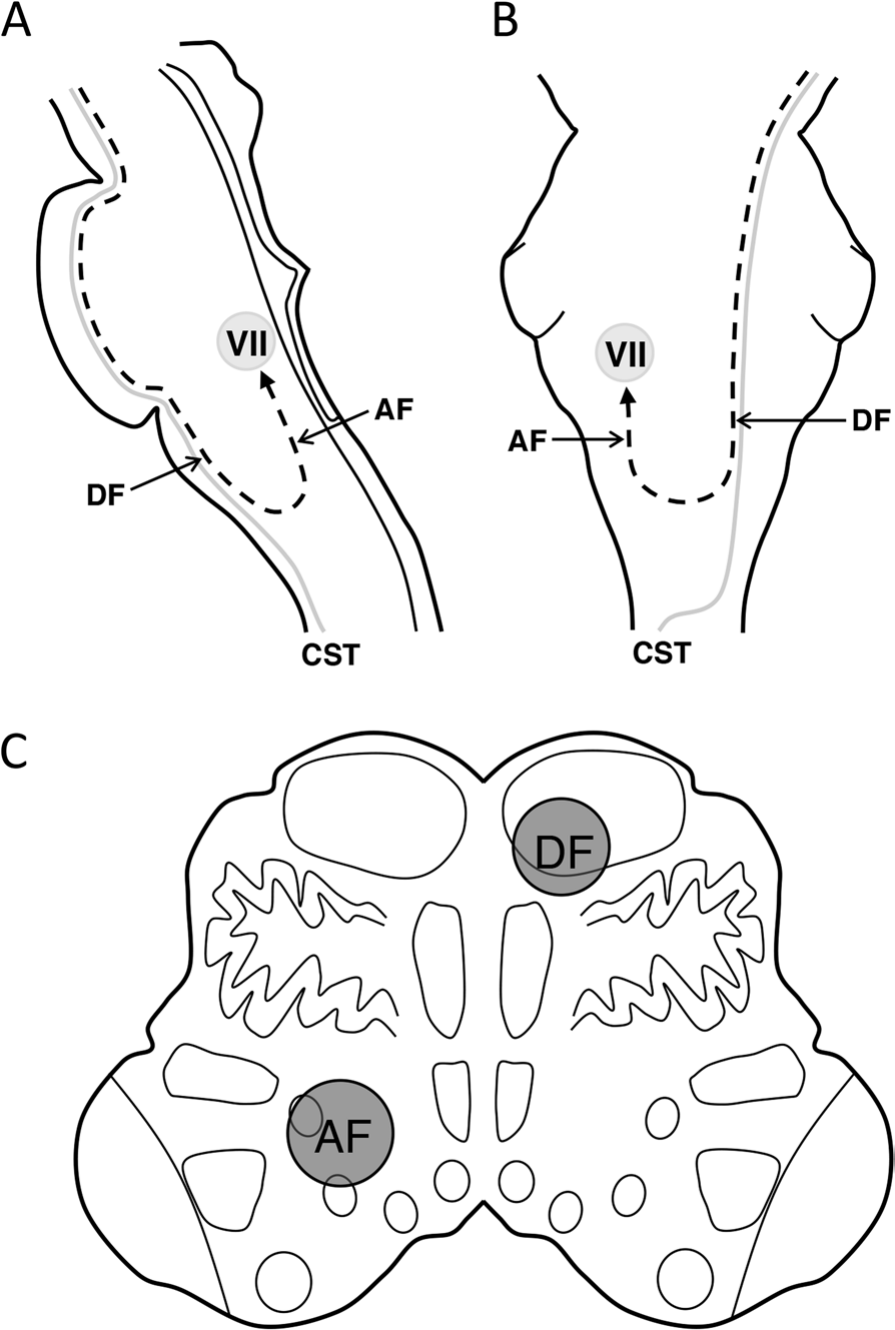
Schematic representation of the course of the corticobulbar tract to the facial nucleus that descend to the level of the medulla oblongata. **a** Sagittal section. **b** Coronal section. **c** Cross-section at the level of the inferior olivary nucleus. The corticobulbar tract (dotted line) to the facial nucleus descends along with the corticospinal tract (CST) to the medulla. Then, after forming a loop and decussation, it ascends through the medial and ventral areas of the dorsolateral medulla to the facial nucleus in the caudal pons. AF, ascending fibers; CST, corticospinal tract; DF, descending fibers

**Fig. 4:**
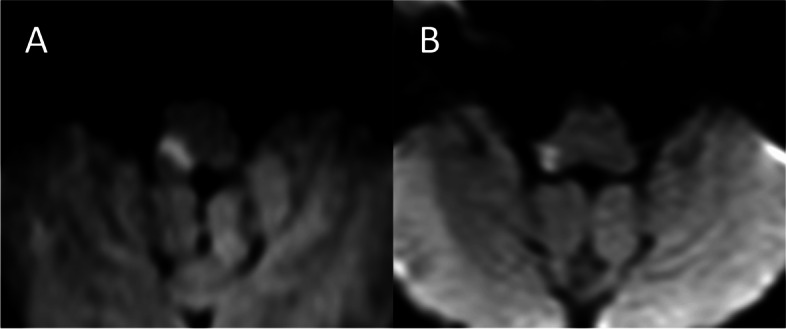
Typical diffusion-weighted images of the middle medullary lesion in patients with and without central facial paresis. **a** The lesion was classified as the ventral type, and the patient presented with central facial paresis on the same side as the lesion. **b** The lesion was classified as the dorsal type, and the patient did not present with central facial paresis

In the horizontal lesion classification in this study, there were no patients with central FP associated with dorsal or lateral types, whereas all the patients with central FP had lesions including the area of the ventral type lesions. Based on these results, facial CBT fibers were considered to ascend through the ventral and medial parts of the dorsolateral medulla (Fig. 3). Figure 4 shows the representative brain MRI findings of the middle medullary lesion in patients with and without central FP (Fig. 4).

In this study, four patients with lesions of the upper medulla and one patient with lesions of the upper to middle medulla did not present with central FP. Among these patients, except for the two patients who presented with peripheral FP, the remaining three had lesions of the dorsal type, i.e., not including the area of the ventral type. When the lesion site is away from the course of the CBT, central FP does not occur even in the lesions of the upper medulla.

## Conclusions

Precise knowledge about the course of the CBT to the facial nucleus can facilitate the identification of localization of the lesions based on neurological examinations. Our results showed that patients with LMI who have ventral and medial lesions may often present with central FP on the same side as the lesion. Moreover, this condition may be significantly associated with dysphagia and hiccups.

## Data Availability

The datasets used and/or analysed during the current study are available from the corresponding author on reasonable request.

## Abbreviations

CBT: Corticobulbar tract
CST: Corticospinal tract
FP: Facial paresis
LMI: Lateral medullary infarction
MRI: Magnetic resonance imaging
